# Warm perches: a novel approach for reducing cold stress effect on production, plasma hormones, and immunity in laying hens

**DOI:** 10.1016/j.psj.2021.101294

**Published:** 2021-05-29

**Authors:** J.Y. Hu, H.W. Cheng

**Affiliations:** ⁎ Department of Animal Sciences, Purdue University, West Lafayette IN 47907, USA; † USDA-Agricultural Research Service, Livestock Behavior Research Unit, West Lafayette, IN 47907, USA

**Keywords:** laying hen, cold stress, warm perch, performance, physiological homeostasis

## Abstract

Cold temperature is a common environmental stressor that induces pathophysiological stress in birds with profound economic losses. Current methods used for preventing cold stress, such as reducing ventilation and using gas heaters, are facing challenges due to poor indoor air quality and deleterious effects on bird and caretaker health. The aim of this study was to examine if the novel designed warmed perch system, as a thermal device, can reduce cold stress-associated adverse effects on laying hens. Seventy-two 32-week-old DeKalb hens were randomly assigned to 36 cages arranged to 3 banks. The banks were assigned to 1 of 3 treatments: cages with warmed perches (**WP**; perches with circulating water at 30°C), air perches (AP, regular perches only), or no perches (**NP**) for a 21-d trial. The room temperature was set at 10°C during the entire experimental period. Rectal temperature and body weight were measured from the same bird of each cage at d 1, 8, 15, and 21 during the cold exposure. Egg production was recorded daily. Feed intake, egg and eggshell quality were determined during the 1st and 3rd wk of cold stress. Plasma levels of corticosterone, thyroid hormones (3, 3’, 5-triiodothyronine and thyroxine), interleukin (**IL**)-6 and IL-10, were determined after 1 d and 21 d of cold exposure. Compared to both AP and NP hens, WP hens were able to maintain their body temperature without increasing feed intake and losing BW. The eggs from WP hens had thicker eggshell during the 3rd wk of cold exposure. Warmed perch hens also had a lower thyroxine conversion rate (3, 3’, 5-triiodothyronine/thyroxine) at d 1, while higher plasma concentrations of IL-6 at d 21. Plasma levels of corticosterone, 3, 3’, 5-triiodothyronine, and IL-10 were not different among treatments. Our results indicate that the warmed perch system can be used as a novel thermal device for preventing cold stress-induced negative effects on hen health and welfare through regulating immunity and metabolic hormonal homeostasis.

## INTRODUCTION

Global climate change has caused frequent and intense heat waves, but also led to extremely low temperatures during the winter months. Intense cold in animals increases feed intake, compromises their health and production, which further results in economic losses (Chen et al., 2012; van Iaer et al., 2014; Nguyen et al., 2016). Commercial laying hens have been genetically selected for high production with low body mass to increase feed conversion ratio. Egg production is energy demanding (Sakomura et al., 2019), together with the low body mass, commercial laying hens’ performance could be worse in cold winter weather as compared to hot summer seasons. It has been estimated by environmental and heat-transference data that laying hens lost 4 times more energy during cold weather to maintain their body temperature (Alves et al., 2012). The deleterious effect of cold stress on hens can be even more severe toward the end-of-lay when the hens have regularly lost a considerable amount of feather coverage (Beaulac et al., 2020).

Previous research conducted in poultry has revealed that acute cold stress suppresses the development, survivability, and production performance in birds (Sahin et al., 2003; Hulzebosch, 2006). For example, mortality due to immunosuppression and ascites is greatly increased in broiler chickens exposed to cold stress. Cold ambient temperatures increase the oxygen requirement and cardiac output, together with peripheral vasoconstriction, leading to increased pulmonary arterial pressure (Ipeck and Sahan; 2006; Qureshi et al., 2018, Alba et al., 2019). In addition, to reduce increased costs associated with feed and heating budgets during winter, poultry producers often close ventilation down and increase the barn temperature set point to reduce feed intake by birds, which in turn facilitates the ammonia (**NH_3_**) emissions and dust built up inside the poultry houses. Ammonia level in laying house should be controlled under 10 ppm without exceeded 25 ppm (UEP, 2017), however, NH_3_ levels have been reported to be as high as from 40 to 100 ppm (Ni et al., 2012) or 70 to 120 ppm (Liang et al., 2005), during winter seasons based on the type of houses. High NH_3_ concentrations adversely affect the health and production of hens as well as animal caretakers (Xin et al., 2011; Zhao et al., 2013), causing respiratory illness including coughing, upper respiratory tract bleeding, excessive secretions, and lung inflammation or infection (Kilic and Yaslioglu, 2014).

Chickens, like other homeothermic animals, maintain their core body temperature without extra effort within their thermoneutral zone, 16 to 23°C. When the ambient temperature climbs above the upper or drops below the lower limit of the thermoneutral zone, heat exchange processes are induced between the body and the ambient environment (Van Kampen et al., 1979). During cold exposure, for example, the hypothalamus, as the thermoregulation center, initiates heat-retention responses, which includes cutaneous vasoconstriction and a variety of behavioral responses, reducing respiration rate, piloerection of feathers, and shivering to conserve or produce body heat (Boulant, 1981; Zhao et al., 2017). In addition, cold stress disrupts biological homeostasis, impairing hormonal and immune functions. In response to stress, the hypothalamic-pituitary-adrenal (**HPA**) axis increases the release of corticosterone (**CORT**) from the adrenal glands, to promote gluconeogenesis in the tissues and lipolysis in the fat, by which CORT enhances glucose availability and energy metabolism (Schweiger et al., 2006; de Kloet and Herman, 2018). Increased plasma levels of CORT have been reported in young broilers chickens (Borsoi et al., 2015) and male turkeys (El-Halawani et al., 1973) under cold exposure. In addition, both 3, 3’, 5-triiodothyronine (**T3**) and thyroxine (**T4**) are the most important thyroid hormones regulating energy metabolism for maintaining thermogenetic homeostasis (Klandorf et al., 1981; Decuypere et al., 2005). Exposing animals to low environmental temperature is associated with increased conversion of T4 to T3, which contributes to increased heat production (Galton and Nisula, 1969; Hangalapura et al., 2004). Furthermore, cold stress causes immunosuppression in animals by exacerbating pathophysiological conditions including rodents (Rastogi and Haldar, 2020) and poultry (Gross and Siegel, 1988; Regnier and Kelley, 1981). Immunosuppression causes changes of immunity and associated cytokines, such as proinflammatory interleukin 6 and anti-inflammatory IL-10 (Lin and Karin, 2007).

Egg laying strains of chickens are highly motivated to perch (Lambe and Scott, 1998; Olsson and Keeling, 2002); they exhibit signs of frustration and restlessness if perching opportunity is thwarted (Olsson and Keeling, 2000). Richly vascularized chicken feet and shanks are functionally as effective conductors to release/gain heat to/from the environment under hot/cold weather. During cold weather, the countercurrent heat exchange enables the blood to be warmed on its way returned to the heart through the superficial veins of the limbs (Dawson and Whittow, 2000). In chickens, more than 25% of metabolic heat can be transferred or regulated through their feet at thermoneutral temperatures (Hillman and Scott, 1989). Based on the empirical heat transfer theory, we hypothesize that laying hens may be benefited during cold exposure by providing them with warmed perches for roosting. The objective of this study was to determine if the newly designed warmed perch system, as a thermal device, can ameliorate the deleterious effects of cold stress in caged laying hens by assessing performance, egg quality, innate immunity cytokines, and the levels of CORT, and thyroid hormones.

## MATERIALS AND METHODS

### Birds and Experimental Design

The study was conducted in February 2019. All hens used in this experiment were housed and handled under the protocol approved by the Animal Care and Use Committee of Purdue University (PACUC#: 1712001657).

At 32 wk of age, 72 DeKalb Laying hens were randomly housed in 2-birds cages (987 cm^2^/hen; n = 36) arranged in 3 banks, and then assigned to 1 of 3 treatments: 1) warm perch (**WP**, perches with circulating warm water, 30°C), 2) air perch (**AP**, regular perches only), and 3) no perch (**NP**) inside a temperature-control, light-tight room for a 21-d (3 wk) trail. Each cage provided: wire mesh floor, 2 nipple drinkers, 12.6 cm feeder space/hen, as well as 38 cm perch space/hen in perch groups (WP and AP). The perch space/hen used in the current experiment (38 cm) provides enough space for both hens to perch simultaneously without crowding or competition. All 3 banks were placed inside of a temperature-control, light-tight room (13 feet wide by 53 feet long). A laying diet with 18.3% CP, 2,890 kcal ME/kg, 4.2% Ca, and 0.3% nonphytate phosphorus was fed to the chickens (Hendrix Genetics, 2020). Hens had free access to food and water throughout the experiment. The lighting schedule was set at 16L: 8D.

### Cold Stress Treatment, Warmed Perch System Design, and Temperature Monitor

Cold stress was set at 10°C and the ventilation system was fully operated throughout the entire experimental period. The thermostat for the heater was set at 10°C to turn on the heating system if outside temperature was too low to maintain the room temperature close to 10°C.

**Figure 1:**
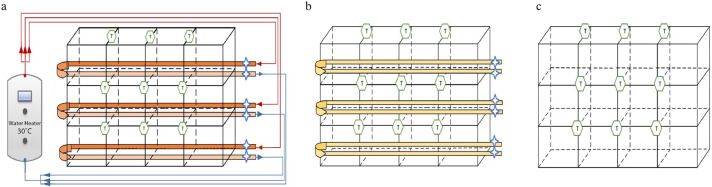
Cage bank design for the 3 treatments: warm perch (WP) cages (A), air perch (AP) cages (B), and control cages with no perches (NP) (C). Two perches were installed parallel in each row of a cage bank. For the WP cages, an electric water heater was used to supply warm water (30°C) to each row. The figure illustrates temperature recording locations (✩) for measuring the water/air temperature at the warm perch and air perch (nonwarmed perch). Air temperature and relative humidity () within each treatment and bank level (top, middle, and bottom) of the cages and in the room will be continuously recorded.

A bank consisted of 3 rows with 4 cages per row. In the perch groups (WP and AP), 2 perches (38 cm/hen) were connected to form a continuous loop per row. An electric water heater (Model: 6EP20-1, Richmond, Atlanta, GA) was used to supply warm water (temperature setpoint: 30°C) for WP cages; and warm water was continuously circulating within each of loops via individual pumps during the 3 wk cold stress period. The AP cages had the same perch setting but without warm water circulating. The temperature and relative humidity (**T/RH**) data loggers (model ZW-007 for cages with perches and model ZW-003 for cages without perches, Onset Computer Co., Bourne, MA) were installed in each row. Two resistance temperature detectors (**RTD**) were installed in each of the warm perch loops to measure the supply and return water temperature (Figure 1a). Similarly, a single point for the air perch was also measured (Figure 1b).

Both the room and cage temperature and room relative humidity were measured and recorded at 1-min intervals throughout the experimental period. Temperature and relative humidity data were averaged every 10 min for analysis.

### Physical and Physiological Sampling

Body weight and rectal temperature were measured from the leg band tagged bird (randomly taken) per cage at d 1 and then weekly during cold stress. Rectal temperature was determined using a digital thermometer (Model: TM99A, Cooper Atkins, Middlefield, CT).

The thermography of the feet was collected with an infrared camera (FLIR T440, FLIR systems, Inc., Wilsonville, OR). The camera was set at an emissivity of 0.95. Two images per bird were taken weekly (AP and WP birds’ feet pictures were taken when they were roosting on the perches). The images were analyzed using the FLIR Tools software (FLIR system Inc.); and the data were averaged for statistical analysis.

Feed intake of each cage was determined over 7 consecutive d during the 1st and 3rd wk during cold stress. The amount of feed was weighed before adding to the trough, and the feed left in the trough and bucket was weighted at the end of each analysis wk. The average daily feed intake per hen was calculated as: (Total kg feed in - kg feed left)/7 d/2 birds (Hu et al., 2019b).

A 5 mL blood sample was taken from the untagged bird per cage (n = 12 hens per treatment) via the brachial vein within 2 min of being handled at both d 1 (24 h after cold exposure) and d 21 during cold stress. To minimize any variation that might be caused by sampling time, the samples were collected at the same time on each sampling day, and during sample collection, one hen was sampled from each treatment consecutively until all 36 hens had been sampled. Blood samples were centrifuged at 700 × g for 20 min at 4°C. The supernatant plasma was collected and stored at -80°C until analysis.

### Behavioral Observation

Hen perching behavior was collected twice daily using live observation at 0900-1000 and 1600-1700 h. Perching behavior was recorded when a bird's both feet are on the perch. From each time point, the same trained observer walked through the room 4 times to count the number of hens perching. The proportion of hens perching were averaged per cage weekly for data analyses.

### Egg Production and Quality

Egg production was recorded daily during the experimental period. Hen-day egg production was calculated as: the total number of eggs produced per cage/number of hens per cage × 100. Four intact hard-shelled eggs were randomly collected from each cage (48 eggs/treatment) during 3 consecutive d at the 1st (1–3 d after cold exposure) and 3rd wk (15–17 d after cold exposure), respectively. Each collected egg was weighed and measured for breaking force individually. The eggs were then frozen to crack the shells. The yolk and albumen were removed from each egg after thawing and discarded. The shell with intact shell membranes was rinsed with water and dried at 60°C. The dried shell weight was recorded. Shell thickness and % shell mass were determined as described by Hu et al. (2019b).

### Plasma Corticosterone

Plasma CORT concentrations were measured using a commercially available radioimmunoassay kit (Catalog #: 07120103, MP Biomedicals, Solon, OH). Total plasma CORT concentrations were analyzed in duplicate with CV ≤15% following the protocol described by Cheng et al. (2001). Both intra- and interassay coefficients of variation were below 10%.

### Plasma Thyroid Hormones

Plasma concentrations of T3 (Catalog #: 06B-254216, MP Biomedicals, Solon, OH) and T4 (Catalog #: 06B-254030, MP Biomedicals, Solon, OH) were analyzed with the commercially available ^I125^RIA kits by following the company's protocols. Briefly, the mixture of 100 µl of T3 standard or plasma sample with T3 tracer was incubated in a water bath at 37 °C for 60 min. At the end of the incubation, all tubes were vacated, and the radioactivity was counted at 1 min per sample with a gamma counter (1470 Wizard Gamma Counter, PerkinElmer, Waltham, MA). A similar procedure was used for T4 analysis except that the counting time was 30 sec per sample. T3/T4 ratio was calculated for each sample. All samples were measured in duplicate with CV ≤ 15%, and the intra- and interassay coefficients of variation were both below 10% (Hu et al., 2019a; Yan et al., 2020).

### Plasma Cytokines Interleukin-6, and Interleukin-10

Plasma concentrations of IL-6 and IL-10 (Catalog #: MBS037319, MBS007312, MyBioSource, San Diego, CA) at d 1 and d 21 post cold stress initiation were analyzed with the commercially available ELISA kits by following the company's protocols. All samples were measured in duplicate with CV ≤15%, and the intra- and interassay coefficients of variation were both less than 15% (Hu et al., 2019a; Yan et al., 2020).

### Statistical Analysis

Data from the randomized design were subjected to an ANOVA using the GLIMMIX procedure for perching behavior analysis or MIXED method for other parameters of SAS 9.4 software (SAS Institute Inc., Cary, NC). Repeated measures were used for performance and physiological traits with row of cages within treatment serving as the experimental unit. Each of the 4 cages within a row of a treatment served as a subsample. Fixed effects were treatment and age of the hens. Subsampling error terms included cages within row and hens within cages within a row. Pooling of error terms occurred when *P* > 0.25. Egg production data were arcsine square root transformed. If data lacked homogenous variances, BOXCOX was used for transformation, and the data were reanalyzed. Because statistical trends were similar for both transformed and untransformed data, the untransformed results are presented. Tukey-Kramer was used to partition differences among means due to significant treatment effects (Steel et al., 1997). The SLICE option was used for the 2-way interaction of treatment and age (Winer et al., 1991). Significant statistical differences were reported when *P* ≤ 0.05, a trend was reported when 0.05 < *P* ≤ 0.1.

## RESULTS

**Figure 2:**
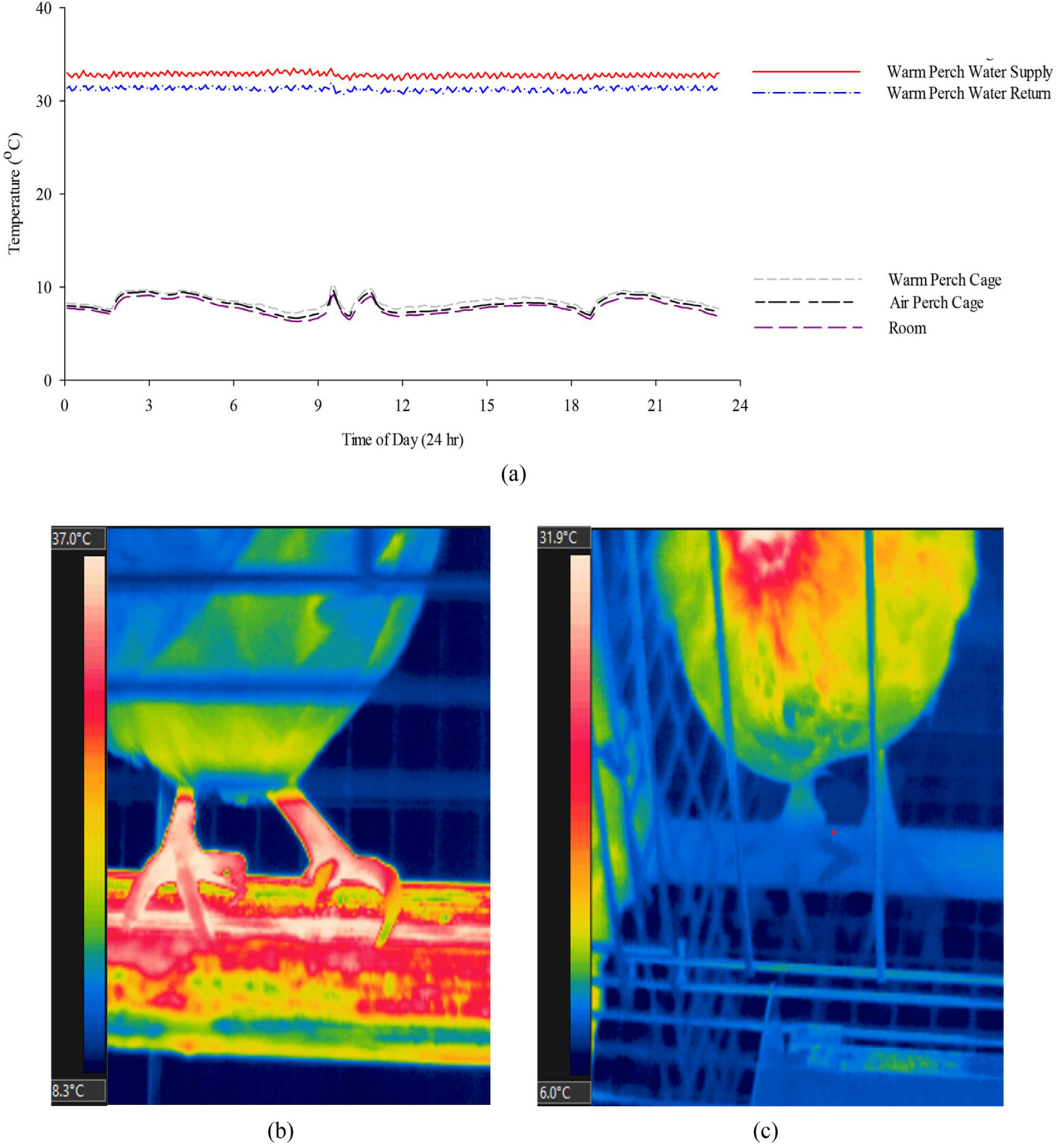
An example of the treatments’ temperatures recorded for 24 h during cold exposure (0000 to 2400 h, from top to bottom, line 1: warm perch (WP) supply water temperature, line 2: warm perch (WP) return water temperature, line 3: warm perch (WP) cage temperature, line 4: air perch (AP) cage temperature, line 5: room temperature (RT) (A), and examples of the feet thermographic images of a hen perching on a warmed perch (B) and on an air perch (C).

The average daily room ambient temperature was at 11.24°C (6.65–17.25°C), and room relative humidity was at an average of 49% (30–67%) throughout the entire experimental period. A typical day room temperature and perch temperatures are shown in Figure 2a. The WP successfully provided a warm source for the hens as indicated by the much higher perch temperature compared to the cage, room, and AP temperature (Figure 2a). The feet temperature of hens standing on WP was higher (Figure 2b) than the hens roosting on the AP (Figure 2c) (*P_treatment_* < 0.0001, Table 1). Higher proportion of WP birds exhibited perching behavior than AP birds (*P*_treatment_ = 0.03, Table 1).

**Table 1:** Effects of warm perches on body temperature, feed intake, BW loss, production, and behavior in cold stress laying hens.

Treatment	Rectal temperature (°C)^1^	Foot surface temperature^2^ (°C)	Feed intake (g)	Body weight Loss^3^ (%)^2^	Hen-day egg production (%)^3^	Proportion of hen perching (%)^4^
AP	40.66^a^	18.39^b^	133.9^AB^	10.02^A^	87.8	29.21
NP	40.29^b^	13.01^c^	141.7^A^	8.02^AB^	84.9	35.14
WP	40.64^a^	29.14^a^	121.3^B^	5.25^B^	93.6	–
n^5^	12	24	12	12	12	12
*P*-Value						
SEM	0.005	1.23	6.05	1.48	4.0	2.00
*P*_treatment_	<0.0001	<0.0001	0.06	0.09	0.33	0.04
*P*_age_	<0.0001	0.0003	0.0002	0.61	0.04	0.96
*P*_treatment*age_	0.98	0.77	0.94	0.99	0.84	0.55

a,b Least squares means within a column for the 3 treatments lacking a common superscript differ (*P* < 0.05).

B Least square means represents a trend among the 3 treatments (0.05 < *P* ≤ 0.1).

1 Values within a column represent the least squares means averaged over 4 measured time points (d 1, 8, 15, and 21 after initiation of cold stress).

2 Values within a column represent the least squares means averaged over 3 measured time points (Weekly after initiation of cold stress).

3 Body weight change was calculated as measured body weight/original body weight*100%.

4 Proportion of hen perch was calculated as the number of perching hens observed/2 hens per cage*100%

5 Average number of observations per least squares mean.

**Figure 3:**
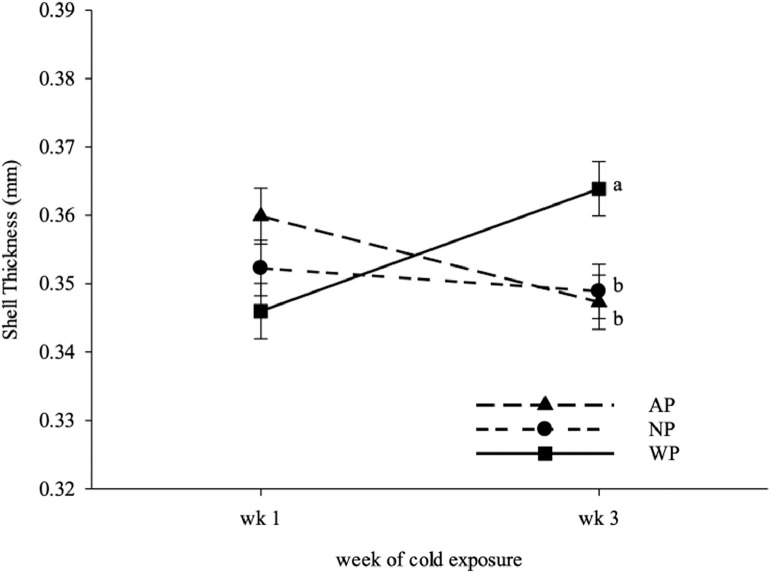
The shell thickness of eggs collected from DeKalb hens submitted to 1 of 3 treatments (▲= air perch or AP, ● = control or NP, ▪ = warm perch or WP) between the 1st and 3rd wk of cold exposure. ^a,b^ Within an age, least squares means ± SEM with no common letter are significantly different (treatment × age interaction, *P* = 0.03).

Rectal temperature was higher in both WP and AP hens than NP hens (*P*_treatment_ < 0.0001, Table 1). Foot surface temperature was warmer in WP hens than NP hens, and AP hens were intermediate (*P*_treatment_ < 0.0001, Table 1). WP hens tended to have lower feed intake than NP but not AP hens (*P*_treatment_ = 0.06, Table 1). The BW loss had a trend to be lower in WP hens than AP hens but not NP hens (*P*_treatment_ = 0.09, Table 1). Egg production was not affected by the treatments (*P* > 0.05) but was reduced as cold stress persisted over the course of the experiment (*P*_age_ = 0.04, Table 2). There was no treatment effect on egg weight, egg breaking force, shell thickness, and shell percentage (*P* > 0.05; Table 2) except that the eggs from WP group during the 3rd wk of cold exposure had thicker shell than both AP and NP hens (*P*_treatment*age_ = 0.03, Table 2, Figure 3).

**Table 2:** Effects of warm perch on egg and eggshell quality in cold stressed laying hens.

Treatment	Egg weight^1^ (g)	Breaking force^1^ (N)	Shell thickness^1^ (mm)	Shell percentage^1^ (%)
AP	55.82	42.07	0.35	9.35
NP	56.89	44.80	0.35	9.27
WP	55.51	44.14	0.35	9.49
n^2^	96	96	96	96
*P*-value				
SEM	0.50	1.19	0.004	0.11
*P*_treatment_	0.13	0.21	0.75	0.37
*P*_age_	0.47	0.52	0.89	0.75
*P*_treatment*age_	0.74	0.13	0.03	0.14

1 Values within a column represent the least squares means of 4 eggs from each of the 3 treatments averaged over 2 ages.

2 Values within a column represent the least squares means averaged over 3 wk of egg production (32 to 34 wk). ^3^Average number of observations per least squares mean.

**Figure 4:**
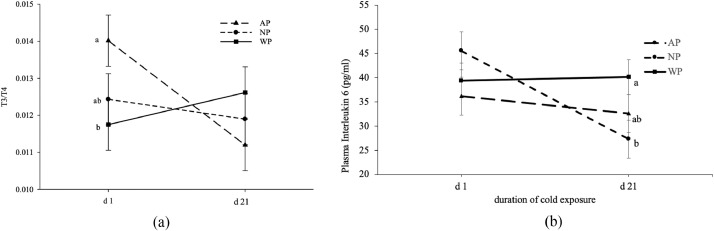
The T3/T4 ratio (A) and plasma interleukin (IL)-6 (B) of DeKalb hens submitted to 1 of 3 treatments (▲= air perch or AP, ● = control or NP, warm perch or WP, ∎ = warm perch or WP) between the 1 d and 3rd wk of cold exposure. ^a,b^ Within an age, least squares means ± SEM with no common letter are significantly different (treatment × age interaction, *P* = 0.05).

Plasma concentrations of CORT, T3, and IL-10 were not different among treatments; while T4 levels were higher in both WP and NP hens than AP hens (*P*_treatment_ = 0.002, Table 3). T3/T4 ratio was lower in WP hens than AP hens after 1 d of cold exposure (*P*_treatment*age_ = 0.05, Table 3, Figure 4a), while NP hens were intermediate. Plasma IL-6 level was higher in WP than NP hens but not AP hens at 21 d of cold exposure (*P*_treatment*age_ = 0.04, Table 3, Figure 4b).

**Table 3:** Effects of warm perch on the levels of corticosterone, thyroid hormones, and cytokines in cold stressed laying hens.

Treatment	CORT (ng/mL)^1^^,^^2^	T3 (ng/dl)^1^^,^^2^	T4 (ug/dl)^1^^,^^2^	T3/T4	IL-6 (pg/mL)^1^^,^^2^	IL-10 (pg/mL)^1^^,^^2^
AP	6.68	160.36	12.76^b^	0.013	34.43	90.16
NP	6.13	164.82	13.67^a^	0.012	36.46	94.27
WP	5.74	173.25	14.23^a^	0.012	42.15	88.35
n^3^	12	12	12	12	12	12
SEM	0.72	7.21	0.79	0.0004	4.13	8.56
*P*-value						
*P*_treatment_	0.65	0.46	0.002	0.69	0.43	0.88
*P*_age_	0.12	0.81	0.03	0.21	0.08	0.001
*P*_treatment*age_	0.07	0.07	0.23	0.05	0.04	0.26

ab Least squares means within a column for the 3 treatments lacking a common superscript differ (*P* < 0.05).

1 CORT: Corticosterone; T3: 3,3’,5-triiodothyronine; T4: Thyroxine; IL-6: interleukin 6; IL-10: interleukin 10.

2 Values within a column represent the least squares means averaged over 2 measurements (d 1 and d 21 of cold stress).

3 Average number of observations per least squares mean.

## DISCUSSIONS

The results from the current study indicated that provision of WP for laying hens has beneficial effects on maintaining body temperature and feed intake without compromising BW, egg production, and egg quality under cold conditions. Generally, when animals are exposed to cold environment, the activated hypothalamic thermoregulatory center increases thermogenesis via vasoconstriction and various endocrine mechanisms for conserving heat loss to maintain core body temperature (Robertshaw, 2006; Collier et al., 2019; Habibu et al., 2019). Additionally, the countercurrent heat exchange and arteriovenous anastomoses (**AVAs**) systems enable avian species to have their feet in contact with a cold surface at high endogenous heat production and energy costs (Hillman et al., 1982; Midtgard, 1989). Under the thermoneutral conditions, chicken maintains their body core temperature within a narrow range from 40.56 to 41.67°C. In the current study, compared to NP hens, both WP and AP hens were able to maintain their core temperature, but the underlying mechanisms could be different. In WP hens, when they are contacting with a heat source (i.e., the perches with circulating water at 30°C), they can receive extra heat from the warmed perches, which is beneficial for them to maintain core body temperature; and this is evidenced by the warmest foot surface temperature determined in the WP hens among the 3 groups. In AP group, the higher body core temperature observed could be due to adjusted metabolic heat production during digestion (Chepete and Xin, 2004; Lu et al., 2017) and fat-burning during lipolysis and perching movement (Gebhardt-Henrich et al., 2017), which is supported by approximately 10% of their body weight loss after 3 wk of cold exposure.

Birds derive most of their energy from food intake. In order to cope with the heat loss under cold weather, birds spontaneously consume excessive feed to maintain the energy required (Ferket and Gernat, 2006) at an estimated 1.5g/1°C per hen (Hulzebosch, 2006). An early study conducted by El-Wailly (1966) indicated that the gross feed intake in zebra finch was linearly increased with decreasing environmental temperature. Similarly, low ambient temperatures in laying hens cause an increase in feed intake, but decreases in egg production, egg quality and feed efficiency (Spinu and Degen, 1993; Kucuk et al., 2006). With the negative effect of cold stress, farmers may suffer from increased feed costs and reduced egg production during the winter seasons, which leads to considerable economic losses. In order to keep hen warm while balancing out the heating and feed costs, farmers usually restrict the ventilation to maintain the ambient temperature. Chickens and related waste generated inside the barns lead to air pollutions, including NH_3_, carbon dioxide, methane, hydrogen sulfide, nitrous oxide gases, and dust (Kocaman et al., 2005; Green et al., 2009). Poor ventilation causes accumulations of these gases to a possible toxic level. Inhalation of gaseous NH_3_ or NH_3_ bound to dust particles can damage the mucous flow and ciliary action in the trachea (Nagaraja et al., 1984; David et al., 2015), leading to great susceptibility of diseases in chickens (Kristensen et al., 2000; Banhazi et al., 2008) as well as respiratory irritation in poultry workers and chicken catchers (Sanderson et al. 1995; Rees et al. 1998). In the current study, under the condition of full ventilation and low ambient temperature (10°C), as compared with the other 2 groups of hens, provision of WP tended to prevent the increase of feed consumption but still able to maintain body weight and egg production rate, by which it may provide benefits without compromising air quality in the poultry house.

Egg production and quality are closely related to circulating calcium (**Ca**) availability. Animals stressed due to environmental temperature are found to affect gastrointestinal (**G**I) function and increase GI permeability (Karl et al., 2018). Moreover, cold stress induced vasoconstriction, which results in an increase in efferent sympathetic activity and a reduction in parasympathetic activity (Manou-Stathopoulou et al., 2015; Greaney et al., 2015, 2017). In chickens, specifically, high blood pressure due to vasoconstriction reduces adequate blood flow to the gut and uterus, further impair nutrients absorption and eggshell calcium deposition (Nolan et al., 1978). Eggs collected from WP hens were not different during the 1st wk of cold exposure but had thicker eggshell than both AP and NP hens during the 3rd wk, indicating installation of WP facilitates the hens to better cope with the low ambient temperature and utilize the Ca during eggshell formation.

Plasma levels of CORT have been used as a marker for evaluating acute physiological coping ability (Kunz-Ebrecht et al., 2003). Stress activates the HPA axis to release glucocorticoids from the adrenal glands. With cold exposure, the acute surge of glucocorticoids enhances lipolysis and attenuates glucose uptake within the adipose tissue in order to cope with the demand for supplying metabolic energy to the brain and other crucial organs (Schweiger et al., 2006; Campbell et al., 2009; Ramage et al., 2016). In poultry, controversial results have been reported regarding plasma CORT in response to cold stress. With various durations of cold exposure and stress levels, an increase of CORT was found in broiler chickens (12°C for 24 d; Olfati et al., 2018), chicks (1.2°C for 3 h; Buckland et al., 1974), and laying hens (0°C for 72 h; Hester et al., 1996). In contrast, Hangalapura et al. (2004) reported a suppressive effect of cold stress on CORT. In the current study, the CORT level was not affected by the treatments. In the current experiment, all the hens were exposed to a cold environment for 3 wk, which could be considered as a chronic stress condition. The unchanged CORT could probably be due to the negative feedback of CORT on the HPA axis with continuously cold exposure, resulting in an inhibition of adrenocorticotropin secretion (Vandernborne et al., 2005). Similarly, previous studies have reported that constantly elevated CORT is not commonly seen under chronic or repeated stress possibly due to the negative feedback of CORT on the HPA axis (Romero, 2002, 2004; Smith and Vale, 2006; Angelier and Wingfield, 2013). The hypothesis will be tested in our future studies.

Thyroid hormones, including both T3 and T4, play important roles in energy metabolism and thermogenesis (Wrutniak-Cabello et al., 2001; McNabb, 2006). Cold exposure has been shown to enhance the conversion from T4 to T3 in order to increase heat production in birds (Burger and Denver, 2002; Collin et al., 2003; Zheng et al., 2013), rodents (Park et al., 2017), and humans (Levine et al., 1995; Bojko, 1997). Birds acclimated to cold temperature have increased thyroid gland size and functional activities than control ones (Faure et al., 2013; McNabb and Darras, 2014). In the current experiment, T4 was found higher in both WP and NP groups than AP group. The provision of WP could allow the chickens to gain heat from the WP, which in turn reduces the need for endogenous heat production from the conversion of T4 to T3, thus leads to higher peripheral T4 determined in WP chickens. For acute cold stress, Hangalapura et al (2004) reported an increased conversion of T4 to T3 after 1 d of cold stress due to a great level of T3, while Rudas and Pethes (1986) indicated there was an acute drop in peripheral T4, resulting in an increase in T3/T4 ratio. Similarly, after 1 d of cold exposure (acute cold stress), we found a lower T3/T4 ratio in WP hens, suggesting the WP installation may possibly reduce the stress intensity and facilitate the chickens with better adaptation to acute cold stimulations. Therefore, the higher T4 found in WP hens could also be considered as an indicator for ameliorated stress response with the provision of WP. While the higher T4 levels found in the NP hens probably resulted from the increased metabolic and digestive heat from excessive feed intake (141.7 g > 133.9 g > 121.3 g; NP > AP > WP).

Prolonged environment stressor exposure can weaken the immune system, leading to immunosuppression. In rodent studies, both acute and chronic cold stress contribute to the immune-suppressive state in the animals (Kizaki et al., 1996; Rykakina et al., 1996; Sesti-Costa et al., 2012). In chickens, studies have reported cold stress decrease the number of lymphocytes, which leads to increased heterophil to lymphocyte ratio which has been used as a stress indicator in various animals including chickens (Hester et al., 1996; Campo et al., 2008; Olfati et al., 2018). In both humans and animals, IL-6 promotes the migration and proliferation of both T and B lymphocytes (Lippitz, 2013). Interleukin-10, as an anti-inflammatory cytokine, has a protective property in inflammation and infection by limiting the immune response to pathogens. Our data have shown the peripheral IL-6 level was higher in WP group than NP hens exposed to cold ambient temperature for 3 wk, which possibly indicates that the prolonged cold stress has contributed to the immunosuppression in NP hens, while the provision of warmed perches could ameliorate the negative effect of low ambient temperature. No difference in IL-10 concentration among the 3 treatments was observed in the current experiment. In contrast to the possible immunosuppressive state found under the current cold stress condition, Hangalapura et al. (2006) reported that cold stress stimulates the immune response, resulting in increased IL-6 but reduced IL-10 concentrations in chickens exposed to cold stress at 10°C for 43 d. In that study, the birds used were 23-day-old when the cold stress was applied. As one of the reasons for the different findings, the immune response to cold stress could be affected differently in chickens as well as in other animals at different ages (Gaunson et al., 2006; Ge et al., 2020).

In conclusion, provision of WP assisted DeKalb laying hens with thermoregulation during the cold exposure as indicated in the measured outcomes including feed consumption, body weight, rectal temperature, foot surface temperature, egg quality traits, thyroid hormones, and immunoregulation. The results show that the warmed perch system could be a novel thermal device for reducing cold stress during the winter seasons. It also provides new sights for examining the effects of the WP system on different growth phases of laying hens under various cold conditions.
